# The miR-34a-LDHA axis regulates glucose metabolism and tumor growth in breast cancer

**DOI:** 10.1038/srep21735

**Published:** 2016-02-23

**Authors:** Xiangsheng Xiao, Xiaojia Huang, Feng Ye, Bo Chen, Cailu Song, Jiahuai Wen, Zhijie Zhang, Guopei Zheng, Hailin Tang, Xiaoming Xie

**Affiliations:** 1 Department of Breast Oncology, Sun Yat-Sen University Cancer Center, State Key Laboratory of Oncology in South China, Collaborative Innovation Center for Cancer Medicine, Guangzhou, 510060, China; 2 Cancer Center of Guangzhou Medical University, Cancer Research Institute of Guangzhou Medical University, Guangzhou Key Laboratory of Translational Medicine on Malignant Tumor Treatment, Guangzhou, 510060, China

## Abstract

Lactate dehydrogenase A (LDHA) is involved in a variety of cancers. The purpose of this study was to investigate the expression, prognostic roles and function of LDHA in breast cancer. We found that LDHA was upregulated in both breast cancer cell lines and clinical specimens using quantitative real-time PCR (qRT-PCR). Immunohistochemistry (IHC) analysis of tissue microarrays (TMAs) showed that high LDHA expression was associated with cell proliferation, metastasis and poor patient overall survival (OS) and disease free survival (DFS). Furthermore, we found that LDHA promoted glycolysis and cell proliferation *in vitro* and *in vivo*. We also performed luciferase reporter assays and found that LDHA was a direct target of miR-34a. Repression of LDHA by miR-34a suppressed glycolysis and cell proliferation in breast cancer cells *in vitro*. Our findings provide clues regarding the role of miR-34a as a tumor suppressor in breast cancer through the inhibition of LDHA both *in vitro* and *in vivo*. Targeting LDHA through miR-34a could be a potential therapeutic strategy in breast cancer.

Breast cancer is one of the most common malignant diseases in women worldwide, and it is estimated that there will be 234,190 new cases and 40,730 deaths in the United States in 2015^1^. As a heterogeneous disease, breast cancer is classified into different categorizations according to its molecular subtype, providing an efficient method for determining appropriate comprehensive therapy and prognosis^2^. In the last few decades, outstanding advances have been made in breast cancer management, including earlier detection and more effective treatments, leading to significant declines in breast cancer deaths and improved outcomes for women living with the disease. However, the prognosis for most patients remains poor^3^. Therefore, it is essential to develop a novel therapeutic strategy for more effective treatment of breast cancer.

MicroRNAs (miRNAs) are endogenous, non-protein-coding, single-stranded 19- to 25-nucleotide RNAs. By binding to the 3′ untranslated regions (UTRs) of targeted mRNAs, miRNAs act as important post-transcriptional regulators^4^. miRNAs play vital roles in fundamental cell processes, such as survival, proliferation, differentiation and apoptosis, and they are crucial for development and metabolism^5^. They also play an important role in the pathogenesis of cancer^6^,^7^,^8^,^9^,^10^. miR-34a has been reported to act as a tumor suppressor miRNA and is always down-regulated in cancers^11^. miR-34a regulates a variety of target mRNAs such as Notch, hepatocyte growth factor receptor (MET), the proto-oncoproteins MYC and MYCN, various cyclins and CDKs, leading to tumor suppression effects such as cell proliferation inhibition, migration and invasion inhibition, cell cycle arrest and apoptosis^11^,^12^,^13^,^14^,^15^,^16^,^17^. Previously, we determined that miR-34a could inhibit the proliferation and migration of breast cancer by targeting B-cell lymphoma 2 (Bcl-2), silent information regulator 1 (SIRT1), E2F transcription factor 3 (E2F3) and CD44^18^,^19^. However, limited information exists regarding the function of miR-34a in breast cancer cell metabolism.

Lactate dehydrogenase A (LDHA) is an enzyme which plays a critical role in the metabolism of tumor cells^20^,^21^,^22^,^23^,^24^. It has been reported that LDHA correlates with the clinicopathologic features and survival outcomes of patients with cancers such as gastric cancer, renal cell carcinoma, pancreatic cancer, esophageal squamous cell carcinoma and colorectal cancer^24^,^25^,^26^,^27^,^28^,^29^,^30^,^31^,^32^. Expression and post-transcriptional modification of LDHA are regulated by several known oncogenes and deacetylases, such as MYC, HIF-1α, forkhead box protein M1 (FOXM1), Krüppel-like factor 4 (KLF4), and SIRT2^23^,^24^,^28^,^33^,^34^,^35^. All of these findings indicate that LDHA could be a novel therapeutic target.

Recently, several miRNAs have been shown to play important roles in metabolism in cancer^36^. However, the mechanisms of miRNAs in glycolysis are unclear. Here, we investigate the roles of LDHA and the association of miR-34a and LDHA in breast cancer.

## Results

### LDHA is upregulated in breast cancer cell lines and clinical specimens

**Figure 1:**
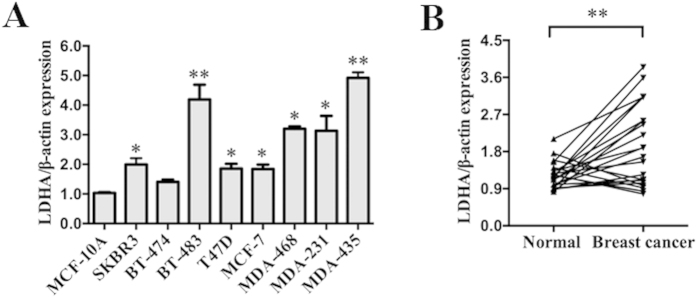
LDHA is upregulated in breast cancer cell lines and clinical specimens. (**A**) Expression levels of LDHA determined by qRT-PCR in nine different mammary cell lines, including one human mammary epithelial cell line (MCF-10A) and eight human breast cancer cell lines. LDHA expression was normalized using β-actin expression. (**B**) Expression levels of LDHA in 22 pairs of breast cancer tissues (Breast cancer) and their matched normal adjacent tissues (Normal). All of the data are shown as the means ± s.e.m. * *P* < 0.05, **P < 0.01.

LDHA is reported to be upregulated in many cancers, including gastric cancer, renal cell carcinoma, pancreatic cancer, esophageal squamous cell carcinoma and others. qRT-PCR analysis was used to detect the expression of LDHA in 9 different mammary cell lines, including the human mammary epithelial (HME) cell line (MCF-10A) and human breast cancer cell lines (SKBR3, BT-474, BT-483, T47D, MCF-7, MDA-MB-468, MDA-MB-231, MDA-MB-435). Compared with HME cell line (MCF-10A), we found that LDHA was upregulated in human breast cancer cell lines, especially in BT-483, MDA-MB-435, MDA-MB-231 and MDA-MB-468 (Fig. 1A). We chose MCF-7 and MDA-MB-231 for further study.

Then, we detected the expression of LDHA in 22 pairs of breast cancer tissues (Breast cancer) and their matched normal adjacent tissues (Normal). Among 22 breast cancer patients, approximately 80% (*P* = 0.0019, 17 of 22 patients) of tumor samples revealed an increase in LDHA expression levels (Fig. 1B). These results indicated a high frequency of upregulation of LDHA in breast cancer and may indicate a relation to the carcinogenesis of breast cancer.

### Increased LDHA level is correlated with cell proliferation, metastasis and poor clinical outcomes

We explored the potential clinicopathological implications arising from altered LDHA levels. The tissue microarrays (TMAs) of 121 breast cancer samples were used for immunohistochemistry (IHC) analysis. The clinical samples were divided into negative or positive expression groups based on LDHA expression (Table 1). We found that LDHA levels were positively correlated with ki67 status and distant metastasis (*P* = 0.013 and 0.001, respectively) but not correlated with age, menopause, tumor size, lymph node metastasis, clinical stage, local relapse, ER, PR, HER2, P53 or VEGF status among the 121 breast cancer patients (Table 1). These results revealed that LDHA might play a vital role in the cell proliferation and progression of breast cancer.

**Figure 2:**
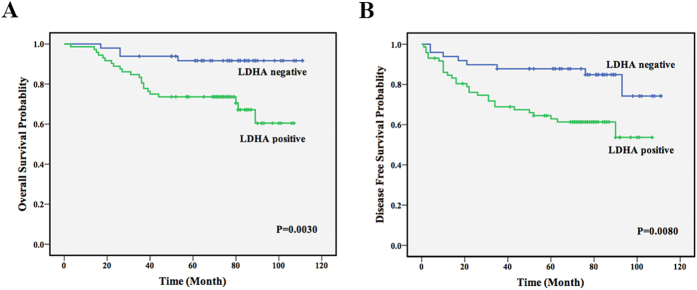
Increased LDHA level is correlated with poor clinical outcomes. **(A**) Positive expression of LDHA correlated with shorter survival. OS curves for 121 studied patients with positive or negative LDHA expression. (**B**). DFS curves for 121 studied patients with positive or negative LDHA expression.

To explore the significance of LDHA in clinical prognosis, we used Kaplan-Meier survival analysis to make the overall survival (OS) and disease-free survival (DFS) curves. The results showed that patients with positive LDHA expression had shorter mean months of OS and DFS than patients with negative expression (*P* = 0.003 for OS, *P* = 0.008 for DFS, Fig. 2A,B). These results demonstrated that positive LDHA expression was significantly associated with poor clinical outcomes.

### LDHA promotes glycolysis and cell proliferation *in vitro* and *in vivo*

**Figure 3:**
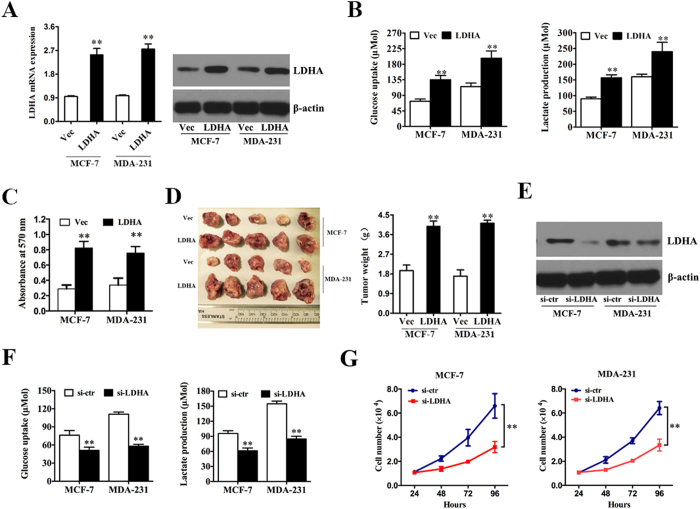
LDHA promotes glycolysis and cell proliferation *in vitro* and *in vivo*. (**A**) MCF-7 and MDA-MB-231 cells were transfected with LDHA-expression vector or control vector. qRT-PCR and western blot analysis demonstrated that the transfection was successful. (**B**) After the transfection, the levels of glucose uptake and lactate production were measured. (**C**) MTT assay was performed after transfection. (**D**) Tumor growth in mouse xenograft models. MCF-7 and MDA-MB-231 cells transfected with LDHA-expression vector or control vector were injected subcutaneously into nude mice (five in each group). After 28 days, the mice were sacrificed, necropsies were performed and then the tumors were weighed. (**E**) MCF-7 and MDA-MB-231 cells were transfected with si-LDHA or si-control. Western blot analysis demonstrated that the transfection was successful. (**F**). After the transfection, the levels of glucose uptake and lactate production were measured. (**E**). Cell numbers were counted after transfection. All of the data are shown as the means ± s.e.m. ** *P* < 0.01.

To assess the biological effects of LDHA in breast cancer, LDHA-expressing vector or control vector was transfected into MCF-7 and MDA-MB-231 cells, respectively. qRT-PCR and western blot analysis demonstrated that the transfection was successful (Fig. 3A). Next, we detected the differences in metabolic parameters and we found that enhanced expression of LDHA largely influenced aerobic glycolysis in breast cancer cells, e.g., increased glucose uptake and lactate production (Fig. 3B). Additionally, when we knocked down the LDHA expression in MCF-7 and MDA-MB-231 cells to confirm the above function of LDHA. Western blot analysis demonstrated that the knockdown of LDHA was successful (Fig. 3E). We detected decreased glucose uptake and lactate production compared with the control group (Fig. 3F). These results indicated that LDHA played vital roles in aerobic glycolysis in breast cancer cells.

To explore and confirm the role of LDHA in breast cancer cells, we performed the proliferation assay in breast cancer cell lines. We found that ectopic expression of LDHA in MCF-7 and MDA-MB-231 cells markedly promoted cell proliferation compared with the control group (Fig. 3C). To further investigate the biological significance of LDHA in tumor growth, xenograft experiments were performed, and we found that ectopic expression of LDHA in MCF-7 and MDA-MB-231 cells led to a significant increase in tumor size and weight (Fig. 3D). But when we knocked down the LDHA expression in MCF-7 and MDA-MB-231 cells, we found decreased cell numbers compared to control group (Fig. 3G). These results indicated that LDHA possessed protumorigenic role in breast cancer cells.

Taken together, these data indicated that LDHA influenced the aerobic glycolysis in breast cancer cells and was essential for breast cancer cell proliferation *in vitro* and *in vivo*.

### LDHA is a direct target of miR-34a

**Figure 4:**
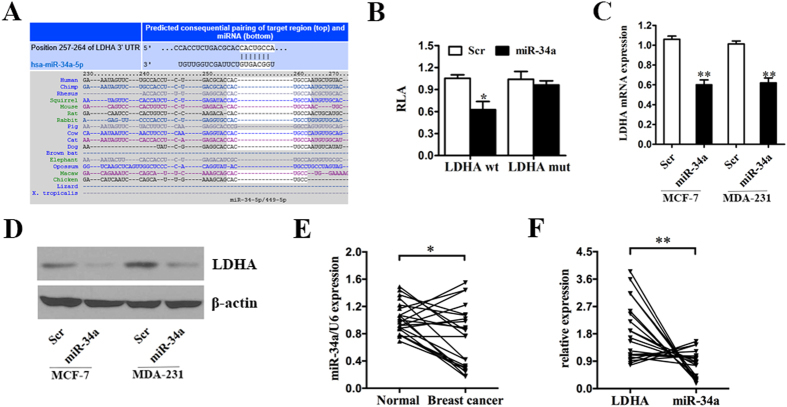
LDHA is a direct target of miR-34a. (**A**) Predicted binding between miR-34a and the seeds matched in the 3′-UTRs of LDHA. (**B**) Luciferase assay of MDA-MB-231 cells cotransfected with miR-34a mimics, scrambled oligonucleotide and a luciferase reporter containing LDHA 3′-UTR (LDHA wt) or mutant constructs in which the first four nucleotides of the miR-34a binding site were mutated (LDHA mut). (**C**) MCF-7 and MDA-MB-231 cells were transfected with miR-34a mimics or scrambled oligonucleotide. miR-34a overexpression inhibited the LDHA mRNA expression. (**D**) MCF-7 and MDA-MB-231 cells were transfected as previously described. miR-34a overexpression inhibited the protein expression of LDHA. β-actin was used as a loading control. (**E**) qRT-PCR was used to detect the expression level of miR-34a in 22 pairs of breast cancer tissues (Breast cancer) and their matched normal adjacent tissues (Normal). (**F**) The correlation between the level of LDHA and miR-34a in 22 breast cancer tissues. All of the data are shown as the means ± s.e.m. *P < 0.05, **P < 0.01.

To identify miRNAs that directly bind to the 3′-UTR of LDHA, we used the mRNA target-predicting algorithms TargetScan and miRanda. Among all the candidate miRNAs, both algorithms predicted miR-34a (Fig. 4A), and we therefore selected miR-34a for further study.

We confirmed this finding by performing luciferase reporter assays in the breast cancer cell line. The full-length LDHA 3′-UTR was cloned downstream of the firefly luciferase gene and co-transfected with miR-34a mimics or the scrambled oligonucleotide. The luciferase activity was measured 48 hours after transfection. The relative luciferase activity of the wild-type 3′-UTR of LDHA in MDA-MB-231 cells in the presence of miR-34a mimics exhibited an approximately 40% reduction in luciferase activity relative to those co-transfected with the scrambled oligonucleotide (Fig. 4B). Additionally, mutation of the putative miR-34a sites in the 3′-UTR of LDHA abrogated the luciferase response to miR-34a (Fig. 4B).

To further confirm that LDHA is a target gene of miR-34a, qRT-PCR and western blot analyses were performed to detect whether the expression of LDHA was regulated by miR-34a in MCF-7 and MDA-MB-231 cells infected with miR-34a mimics or the scrambled oligonucleotide. The result showed a notable reduction in the mRNA and protein levels of LDHA in the cells infected with miR-34a mimics compared with those infected with the scrambled oligonucleotide (Fig. 4C,D).

To test whether the regulations described above for breast cancer cell lines are also clinically relevant, qRT-PCR was used to detect the expression level of miR-34a in 22 pairs of breast cancer tissues (Breast cancer) and their matched normal adjacent tissues (Normal) as previously described. Indeed, we found that the expression levels of miR-34a in approximately 68% (*P* = 0.0152, 15 of 22 patients) of breast cancer tissues were lower compared with the normal tissues (Fig. 4E). Moreover, we analyzed the correlation between the levels of LDHA and miR-34a in 22 breast cancer tissues and detected a negative correlation between them (Fig. 4F).

Together, these results indicated that LDHA was a direct downstream target of miR-34a in breast cancer cells.

### LDHA-induced glycolysis and cell proliferation can be inhibited by miR-34a

**Figure 5:**
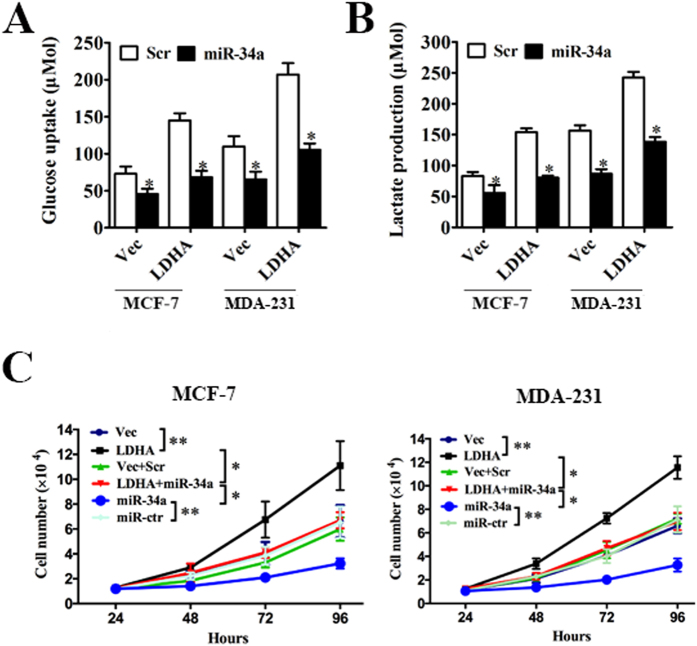
LDHA-induced glycolysis and cell proliferation can be inhibited by miR-34a. (**A**) MCF-7 and MDA-MB-231 cells were transfected with control vector or LDHA-expression vector followed by scrambled oligonucleotide or miR34a mimics. After transfection, the level of glucose uptake was measured. (**B**) MCF-7 and MDA-MB-231 cells were transfected as described before. The level of lactate production was measured after transfection. (**C**) MCF-7 and MDA-MB-231 cells were transfected with control vector, LDHA-expression vector, control vector + scrambled oligonucleotide, LDHA expressing vector + miR-34a mimics, scrambled oligonucleotide or miR-34a mimics. The number of cells was counted. All of the data are shown as the means ± s.e.m. *P < 0.05, **P < 0.01.

The above results prompted us to validate that miR-34a could indeed inhibit the glycolysis and cell proliferation of breast cancer by targeting LDHA. For this purpose, MCF-7 and MDA-MB-231 cells were transfected with control vector + scrambled oligonucleotide, control vector + miR-34a mimics, LDHA-expressing vector + scrambled oligonucleotide, LDHA expressing vector + miR-34a mimics, respectively. The results showed that increased glycolysis induced by LDHA could be repressed by miR-34a, leading to decreased glucose uptake (Fig. 5A) and lactate production (Fig. 5B).

Next, we explored whether miR-34a could inhibit cell proliferation by targeting LDHA. MCF-7 and MDA-MB-231 cells were transfected with control vector, LDHA-expressing vector, control vector + scrambled oligonucleotide, LDHA expressing vector + miR-34a mimics, scrambled oligonucleotide or miR-34a mimics. The results showed that increased expression of LDHA promoted cell proliferation, while increased miR-34a inhibited cell proliferation. However, when transfected with both LDHA expressing vector and miR-34a mimics, increased cell proliferation induced by LDHA was inhibited by miR-34a (Fig. 5C). These results confirmed that miR-34a inhibited cell proliferation through targeting of LDHA.

## Discussion

Breast cancer is a heterogeneous disease with various biological and clinical characteristics. Based on its molecular subtype, different therapies have been developed to better treat the disease. In recent years, outcomes have significantly improved and breast cancer deaths have declined in patients with the disease. However, the prognosis for most patients remains poor. Novel therapeutic strategies are needed to treat breast cancer more effectively.

Reprogramming of energy metabolism, such as elevated glycolysis, is a common feature of cancer due to the high energy demand but low ATP-generating efficiency in cancer cells because of the Warburg effect^37^,^38^,^39^,^40^. Cancer cells increase glucose uptake and metabolic intermediates to support rapid cell growth. Among the glycolytic enzymes, LDH is necessary to maintain high glycolysis rates by producing the NAD + required in the early stages of glycolysis^40^. Advances in the understanding of the progression and metastasis of tumors have clearly highlighted the importance of aberrant tumor metabolism, which supports tumor cells’ energy requirements and their enormous biosynthetic needs. In this study, we explored the critical roles of LDHA in aerobic glycolysis and cell proliferation in breast cancer cells and the mechanism of how miR-34a regulated LDHA and decreased its protein levels and activity. We demonstrated that LDHA upregulation led to increased glucose uptake and lactate production in breast cancer cells and significantly promoted cell proliferation in human breast cancer cell lines both *in vitro* and *in vivo*.

The expression of LDHA is commonly upregulated in cancers^21^,^22^,^24^,^25^,^26^,^30^. In breast cancer cells, we found that miR-34a regulated LDHA levels by targeting the 3′ UTR of LDHA (Fig. 4). The miR-34 family was originally cloned and characterized in 2007 as a p53 target gene^41^. In recent years, the miR-34 family and particularly miR-34a was identified as an important tumor suppressor^11^. In pancreatic cancer, miR-34 suppresses stem cell self-renewal via directly modulating the downstream targets Bcl-2 and Notch^42^. miR-34a negatively regulates CD44 and inhibits prostate cancer regeneration and metastasis^13^. In glioblastoma, miR-34a is identified as a tumor suppressor due to its regulation of the TGF-β signaling network^43^. miR-34a suppresses self-renewal and differentiation by targeting Notch1 in colon cancer stem cells^44^. Previously, our team also found that miR-34a inhibited the proliferation and migration of breast cancer cells by downregulating Bcl-2 and SIRT1^18^, and we have developed the T-VISA-miR-34a which may provide a useful, specific, and safe targeted therapeutic strategy for breast cancer^19^. Due to the important role that miR-34a plays in cancer, the development of miR-34a-based gene therapy is encouraged for multiple types of cancer.

In summary, our study detected a high expression level of LDHA in breast cancer, which was associated with poor clinical outcomes. This finding may be due to the fact that LDHA promoted glycolysis and cell proliferation in breast cancer. We also verified that LDHA is a direct target of miR-34a, and its function in glycolysis and cell proliferation could be inhibited by miR-34a. Our findings provide significant insights into the function of LDHA in breast cancer, and targeting LDHA through miR-34a may be a novel, targeted therapeutic strategy for breast cancer.

## Methods

### Cell lines and culture

The human breast cancer cell lines MCF-7 and MDA-MB-231 were obtained from American Type Culture Collection and cultured in Dulbecco’s modified Eagle’s medium (DMEM, Gibco, USA) supplemented with 10% fetal bovine serum (Gibco) at 37 °C in humidified atmosphere with 5% 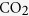.

### Clinical samples

Tissue samples of 22 breast cancer tissues (Breast cancer) and corresponding paired normal adjacent tissues (Normal) were immediately cut and stored in RNAlater (Ambion) and subjected to quantitative real-time PCR (qRT-PCR) analysis. The tissue microarrays (TMAs) consisted of 121 cases of breast cancer tissues diagnosed by histopathological diagnosis from October 2001 to September 2006. Specimens were obtained during surgery and fixed in formalin and embedded in paraffin through standard methods and stored in the Department of Specimens and Resources of Sun Yat-Sen University Cancer Center. This study was approved by the Ethics Committee of Sun Yat-Sen University Cancer Center Health Authority. Informed consent was obtained from all patients. The collection and use of tissues followed procedures that are in accordance with the ethical standards formulated in the Declaration of Helsinki.

### Quantitative RT-PCR analysis (qRT-PCR)

Total cellular RNA was extracted using TRIzol reagent (Invitrogen, USA) according to the manufacturer’s instructions. Reverse transcription and qRT-PCR reactions were performed by means of a qSYBR-green-containing PCR kit (Qiagen, USA). The primers for LDHA were synthesized by Invitrogen: forward, 5′-TTGGTCCAGCGTAACGTGAAC-3′ and reverse, 5′-CCAGGATGTGTAGCCTTTGAG-3′. The threshold cycle (CT) value for LDHA was normalized against the CT value for control β-actin, while U6 snRNA was used as an internal control for the relative amount of miR-34a. The relative fold-change in expression with respect to a control sample was calculated by the 2^−ΔΔCt^ method. All of the real-time PCR assays were performed with the Bio-Rad IQTM5 Multicolour Real-Time PCR Detection System (USA).

### Immunohistochemistry (IHC) analysis

IHC was performed on TMA sections using anti-LDHA antibody (Affinity, USA). The complex was visualized with streptavidin/peroxidase and DAB complex, and the nuclei were counterstained with hematoxylin. The expression status of LDHA was defined as negative or positive according to a previous description^45^.

### LDHA-expressing vector

An LDHA-expressing vector was constructed as follows: the full-length LDHA cDNA was purchased from GeneCopeia TM (USA) and was subcloned into the eukaryotic expression vector pcDNA3.1( + ); the vector pcDNA3.1( + ) was used as a negative control.

### Measurement of glucose consumption and lactate production

Either the LDHA-expressing vector or the control vector was transfected into MCF-7 and MDA-MB-231 breast cancer cell lines. Cell culture media were collected 48 hours after the transfection. Glucose uptake and lactate production were measured using Amplex® Red Glucose/Glucose Oxidase Assay Kit (Invitrogen, USA) and lactate assay kit (Sigma, USA), respectively. The results were normalized on the basis of total cellular protein amounts.

### MTT assay

The cell viability was examined by the MTT assay. MCF-7 and MDA-MB-231 cells were seeded at a density of 5,000 cells per well in 96-well plates and incubated at 37 °C for 24 hours before transfection with either LDHA-expressing vector or control vector. Then, the cells were incubated for another 48 hours, and the MTT assay was performed according to the manufacturer’s instructions (Molecular Probes, USA). The absorbance values were determined at 570 nm using a Spectra Max 250 spectrophotometer (Molecular Devices, USA).

### Mouse xenograft model

A total of 2 × 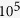 MCF-7 or MDA-MB-231 cells infected with LDHA-expressing vector or control vector were inoculated subcutaneously into the dorsal flanks of nude mice (five in each group). After 28 days, the mice were sacrificed, necropsies were performed, and the tumors were weighed. All of the animal procedures were performed in accordance with institutional guidelines.

### Luciferase reporter assay

The full-length of the 3′-UTR of LDHA was amplified by PCR from MDA-MB-231 genomic DNA and inserted into pGL3 control vector (Promega, WI). Using the QIAGEN XL-site directed Mutagenesis Kit (QIAGEN, CA), we generated several inserts by deletions of 4 bp from the perfectly complementary site of the LDHA gene. According to the manufacturer’s instructions (solution V, program T-016), MDA-MB-231 cells were cotransfected with 0.5 μg firefly luciferase report vector and 0.5 μg control vector containing Renilla luciferase, pRL-TK (Promega) by nucleoporation (AmaxaBiosystems). Each nucleoporation used 50 nM of the miR-34a mimics or scrambled oligonucleotide. 48 hours after transfection, the relative luciferase activities (RLA) of firefly and Renilla were consecutively measured using the dual luciferase assay (Promega).

### Western blot

MCF-7 and MDA-MB-231 cells were transfected with either miR-34a mimics or scrambled oligonucleotide. After 48 hours, protein was extracted from the cell lines using RIPA lysis buffer with a proteinase inhibitor. The protein concentrations in the lysates were measured with the Protein BCA Assay Kit (Bio-Rad, USA), and 20 μg of protein mixed with 2 × SDS loading buffer was loaded per lane. The proteins in the lysates were separated by 12% SDS-polyacrylamide gel electrophoresis and transferred to polyvinylidene difluoride membranes (Millipore, USA). To block nonspecific binding, the membranes were incubated with 5% skim milk powder at room temperature for one hour. Then, the membranes were incubated with an antibody against LDHA (Affinity, USA) for 12 hours at 4 °C. A peroxidase-conjugated secondary antibody (1:400 dilution) and ECL western blotting detection reagents (ECL New England Biolabs, USA) were used to visualize the target proteins, which were quantified with a Bio Image Intelligent Quantifier 1-D (Version 2.2.1, Nihon-BioImage Ltd., Japan). An anti-β-actin antibody (Affinity, USA) was used as a protein loading control.

### Statistical analysis

Comparisons between groups were analyzed with t tests and χ^2^ tests. OS and DFS curves were plotted according to the Kaplan–Meier method, and the log-rank test was used for comparison. Survival was counted from the day of the surgery. All of the differences were statistically significant at the *P* < 0.05 level. The statistical analyses were performed using the SPSS 16.0 software.

## Additional Information

**How to cite this article**: Xiao, X. *et al.* The miR-34a-LDHA axis regulates glucose metabolism and tumor growth in breast cancer. *Sci. Rep.* **6**, 21735; doi: 10.1038/srep21735 (2016).

## Figures and Tables

**Table 1:** Clinicopathological variables and LDHA expression in 121 breast cancer patients.

**Characteristics**	**Total (n = 121)**	**LDHA negative (n = 49)**		**LDHA positive (n = 72)**		
		**No**.	**%**	**No**.	**%**	***P*** **value**
OS						0.003*
Present	95	45	47.4	50	52.6	
Absent	26	4	15.4	22	84.6	
DFS						0.008*
Present	85	41	48.2	44	51.8	
Absent	36	8	22.2	28	77.8	
Age (years)						0.868
< 50	73	30	41.1	43	58.9	
≥ 50	48	19	39.6	29	60.4	
Menopause						0.968
Yes	59	24	40.7	35	59.3	
No	62	25	40.3	37	59.7	
Tumor size (cm)						0.986
≤ 2	32	13	40.6	19	59.4	
> 2	89	36	40.4	53	59.6	
LNMET						0.832
Yes	73	29	39.7	44	60.3	
No	48	20	41.7	28	58.3	
TNM stage						0.196
I-II	63	29	46.0	34	54.0	
III- IV	58	20	34.5	38	65.5	
Local relapse						0.399
Yes	6	1	16.7	5	83.3	
No	115	48	41.7	67	58.3	
Distant metastasis						0.001*
Yes	32	5	15.6	27	84.4	
No	89	44	49.4	45	50.6	
ER status						0.148
Positive	45	22	48.9	23	51.1	
Negative	76	27	35.5	49	64.5	
PR status						0.332
Positive	48	22	45.8	26	54.2	
Negative	73	27	37.0	46	63.0	
HER-2 status						0.711
Positive	18	8	44.4	10	55.6	
Negative	103	41	40.8	62	60.2	
P53 status						0.398
Positive	71	31	43.7	40	56.3	
Negative	50	18	36.0	32	64.0	
VEGF status						0.882
Positive	23	9	39.1	14	60.9	
Negative	98	40	40.8	58	59.2	
Ki67 status						0.013*
Positive	43	11	25.6	32	74.4	
Negative	78	38	48.7	40	51.3	

^*^statistically significant (*P* < 0.05).

% signifies percentage within the row.
